# PB.36: Double reporting of patients discharged from the assessment clinic: the North Lancashire and South Cumbria experience

**DOI:** 10.1186/bcr3536

**Published:** 2013-11-08

**Authors:** G Mataka

**Affiliations:** 1 University Hospitals of Morecambe Bay NHS Foundation Trust, Lancaster, UK; 2 North Lancashire and South Cumbria Breast Screening Programme, Lancaster, UK

## Introduction

There are currently no available guidelines regarding the double reporting of patients discharged from the assessment clinic back to the screening programme.

## Methods

A retrospective audit of patients who were referred for arbitration following discordant double reporting of patients discharged from the assessment clinic was performed. This was performed over a 2-year period from May 2011 to May 2013. The available imaging was reviewed. The outcome of arbitration was documented. The morphology of the lesion, whether a second assessment was required and outcome of the second assessment were also recorded.

## Results

A total of 4,445 patients were assessed in our breast unit. All patients discharged back to the screening programme following assessment were double reported at the end of the clinic. Thirteen (0.29%) patients were referred for arbitration. The morphology of the lesions was as follows: asymmetric density (*n *= 8), microcalcification (*n *= 4) and distortion (*n *= 1). Following arbitration, nine patients were discharged back to the screening programme. The remaining four patients underwent a second assessment. All patients who underwent a second assessment had a biopsy performed. Three of these patients were discharged following a benign biopsy result. The remaining patient was diagnosed with a grade I invasive ductal carcinoma.

## Conclusion

The number of patients requiring a second assessment is very small (0.09%), limiting the psychological impact on our patients. Asymmetric densities are the most common abnormality resulting in referral for arbitration. Double reporting of assessment clinic patients discharged back to the screening programme is now standard practice in our unit.

